# Shiga toxin-producing *Escherichia coli* in human, cattle, and foods. Strategies for detection and control

**DOI:** 10.3389/fcimb.2014.00089

**Published:** 2014-07-02

**Authors:** Nora L. Padola, Analía I. Etcheverría

**Affiliations:** Animal Health and Preventive Medicine, Inmunochemistry and Biotechnology, CIVETAN-CONICET-CICPBA-Faculty of Veterinary Sciences- Universidad Nacional del Centro de la Provincia de Buenos AiresTandil, Buenos Aires, Argentina

**Keywords:** STEC, cattle, food, environment, virulence factors

Shiga toxin-producing *E. coli* (STEC) also known as “verocytotoxin-producing *E. coli*,” refers to *E. coli* pathotypes capable of producing Shiga toxin type 1 (Stx1), type 2 (Stx2), or both, encoded by *stx1* and stx2 genes, respectively (Paton and Paton, 1998). The genes encoding Stx are carried by temperate bacteriophages insert into bacterial genoma so that Stx production is linked to the induction of the phage lytic cycle (O'Loughlin and Robins-Browne, 2001). STx2 is the toxin type most related to hemolytic uremic syndrome (HUS) and comprise several subtypes which differ in their citotoxicity (Persson et al., 2007). Stx2g is one of those subtypes that were studied by Granobles Velandia et al. (2012) who found several differences among *stx*2g-positive strains. The strains with the highest cytotoxic titer showed higher levels of *stx*2-phages and toxin production by EIA, while the opposite occured for strains that previously showed low cytotoxic titers, confirming that in *stx*2g-positive strains Stx production is phage regulated.

Other typical virulence factor is intimin, which is required for intimate bacterial adhesion to epithelial cells inducing a characteristic lesion defined as “attaching and effacing” (A/E). It is encoded by *eae* gene that presents heterogeneity in their 3′ end and involved in binding to the enterocytes (Guth et al., 2010). Additional virulence-associated markers are a plasmid-encoded enterohemolysin and, in strains lacking *eae*, an autoagglutinating adhesin (Saa) which could be involved in the adhesion of strains (Paton et al., 2001). Strains laking *eae* are named as LEE-negative STEC. Steyert et al. (2012) demonstrate that the overall genome content, phage location, and combination of potential virulence factors are variable in this strains group.

STEC are zoonotic pathogens that cause the vascular endothelial damage observed in patients with hemorrhagic colitis (HC) and HUS. HUS is characterized by acute renal failure, thrombocytopenia, and microangiopathic hemolytic anemia and is a potentially fatal cause of acute renal failure in children (Etcheverría and Padola, 2013). HUS there has not treatment and use of antimicrobial agents is associated with an increased risk of severe sequelae such as HUS. Referred to this, Rahal et al. (2012) dicussed novel modalities and regimen of antimicrobial agent administration in an attempt at decreasing their association with aggravating infection outcomes.

Cattle are the main reservoir of STEC and shed the bacteria through their feces spreading these pathogens among cattle herds and the environment. Nguyen and Sperandio (2012) review about the factors and mechanism utilized by O157:H7 STEC for its survival through the acidic environment of the distal stomach and for its colonization in the recto-anal junction. Fernández et al. (2013) characterized two most prevalent serotypes in argentinian cattle demonstrating the potential pathogenic of this strains. Blanco Crivelli et al. (2012) informed that synanthropic species could play role in the transmissibility of the agent thus being a risk to the susceptible population.

Food, water, milk, and person to person contact commonly participate in transmission, although there is a growing concern about some sporadic cases and outbreaks attributable to direct contact with the animal environment (Duffy, 2003). Brusa et al. (2013) report the prevalence of STEC O157 and non-O157 in commercial ground beef and ambient samples, including meat table, knife, meat mincing machine, and manipulator hands suggesting cross-contamination between meat and the environment. One method for reducing STEC in food could be the use of phages. About this, Tomat et al. (2013) inform the isolation of phages highly specific for virotypes of *E. coli* that could be useful in reducing STEC in meat products.

In order to diagnose STEC (O157 and non-O157) several methods have been implemented in the last years (Padola, 2014). Botkin et al. (2012) investigate a multiplex PCR to differentiate EPEC, STEC, and EHEC strains from other pathogenic *E. coli*, Fratamico and Bagi (2012) use a GeneDisc system to evaluate a new PCR-real time technology based on simultaneous detection of multiple targets, Quiñones et al. (2012) evaluate a DNA microarray targeted 12 virulence factors implicated in produce human disease while Parma et al. (2012) developed a sandwich ELISA for determination of Stx using anti-Stx2 B subunit antibodies showing that could be used in routine diagnosis as a rapid, specific and economic method for detection of STEC. The implementation of Multiple-locus variable-number tandem repeat analysis (MLVA) as subtyping method is review by Bustamante et al. (2012) who have adapted this method for analysis of non-O157 STEC performing an efficient O157:H7 and non-O157 STEC subtyping.

## Conflict of interest statement

The authors declare that the research was conducted in the absence of any commercial or financial relationships that could be construed as a potential conflict of interest.

## References

[B1] Blanco Crivelli X. Rumi M. V. Carfagnini J. C. Degregorio O. Bentancor A. (2012). Synanthropic rodents as possible reservoirs of shigatoxigenic *Escherichia coli* strains. Front. Cell. Infect. Microbiol. 2:134 10.3389/fcimb.2012.00134 23125967 PMC3485522

[B2b] Botkin D. J. Galli L. Sankarapani V. Soler M. Rivas M. Torres A. G. (2012). Development of a multiplex PCR assay for detection of Shiga toxin-producing *Escherichia coli*, enterohemorrhagic *E. coli*, and enteropathogenic *E. coli* strains. Front. Cell. Inf. Microbio. 2:8 10.3389/fcimb.2012.00008 22919600 PMC3417533

[B2] Brusa V. Aliverti V. Aliverti F. Ortega Eneas E. de la Torre J. H. Linares L. H. (2013). Shiga toxin-producing *Escherichia coli* in beef retail markets from Argentina. Front. Cell. Infect. Microbiol. 2:171 10.3389/fcimb.2012.00171 23346554 PMC3548221

[B2a] Bustamante A. V. Sanso A. M. Parma A. E. Lucchesi P. M. A. (2012). Subtyping of STEC by MLVA in Argentina. Front. Cell. Inf. Microbio. 2:111 10.3389/fcimb.2012.00111 22919698 PMC3424435

[B3] Duffy G. (2003). Verocytotoxigenic *Escherichia coli* in animal faeces, manures and slurries. J. Appl. Microbiol. 94, 94S–103S 10.1046/j.1365-2672.94.s1.11.x 12675941

[B4] Etcheverría A. Padola N. L. (2013). Shiga toxin-producing *Escherichia coli*: factors involves in virulence and cattle colonization. Virulence 4, 366–372 10.4161/viru.24642 23624795 PMC3714128

[B5] Fernández D. Krüger A. Polifroni R. Bustamante A. Sanso A. M. Etcheverría A. I. (2013). Characterization of Shiga toxin-producing *Escherichia coli* O130:H11 and O178:H19 isolated from dairy cows. Front. Cell. Infect. Microbiol. 3:9 10.3389/fcimb.2013.00009 23483233 PMC3592196

[B6] Fratamico P. Bagi L. K. (2012). Detection of Shiga toxin-producing *Escherichia coli* in ground beef using the genedisc Real-Time PCR system. Front. Cell. Infect. Microbiol. 2:152 10.3389/fcimb.2012.00152 23267438 PMC3526733

[B7] Granobles Velandia C. V. Krüger A. Parma Y. R. Parma A. E. Lucchesi P. M. A. (2012). Differences in Shiga toxin and phage production among stx2g-positive STEC strains. Front. Cell. Infect. Microbiol. 2:82 10.3389/fcimb.2012.00082 22919673 PMC3417572

[B8] Guth B. E. C. Prado V. Rivas M. (2010). Shiga toxin-producing *Escherichia coli*, in Pathogenic Escherichia coli in Latin America, ed Torres A. G. (Washington, DC: Bentham Science Publishers Ltd), 65–83

[B9] O'Loughlin E. V. Robins-Browne R. M. (2001). Effect of Shiga toxin and Shiga-like toxins on eukaryotic cells. Microbes Infect. 3, 493–507 10.1016/S1286-4579(01)01405-8 11377211

[B10] Padola N. L. (2014). Advances in detection methods for Shiga toxin–producing *Escherichia coli* (STEC). Front. Microbiol. 5:277 10.3389/fmicb.2014.00277 24926291 PMC4046177

[B11] Parma Y. R. Chacana P. A. Lucchesi P. M. A. Roge A. Granobles Velandia C. V. Krüger A. (2012). Detection of Shiga toxin-producing *Escherichia coli* by sandwich enzyme-linked immunosorbent assay using chicken egg yolk IgY antibodies. Front. Cell. Infect. Microbiol. 2:84 10.3389/fcimb.2012.00084 22919675 PMC3417390

[B12] Paton A. W. Srimanote P. Woodrow M. C. Paton J. C. (2001). Characterization of Saa, a novel autoagglutinating adhesin produced by locus of enterocyte effacementnegative Shiga-toxigenic *Escherichia coli* strains that are virulent for humans. Infect. Immun. 69, 6999–7009 10.1128/IAI.69.11.6999-7009.2001 11598075 PMC100080

[B13] Paton J. C. Paton A. W. (1998). Pathogenesis and diagnosis of Shiga toxin-producing *Escherichia coli* infections. Clin. Microbiol. Rev. 11, 450–479 9665978 10.1128/cmr.11.3.450 PMC88891

[B14] Persson S. Olsen K. E. P. Ethelberg S. Scheutz F. (2007). Subtyping method for *Escherichia coli* Shiga toxin (verocytotoxin) 2 variants and correlations to clinical manifestations. J. Clin. Microbiol. 45, 2020–2024 10.1128/JCM.02591-06 17446326 PMC1933035

[B14a] Quiñones B. Swimley M. S. Narm K.-E. Patel R. N. Cooley M. B. Mandrell R. E. (2012). O-antigen and virulence profiling of Shiga toxin-producing *Escherichia coli* by a rapid and cost-effective DNA microarray colorimetric method. Front. Cell. Inf. Microbio. 2:61 10.3389/fcimb.2012.00061 22919652 PMC3417394

[B15] Rahal E. A. Kazzi N. Nassar F. J. Matar G. M. (2012). *Escherichia coli* O157:H7-Clinical aspects and novel treatment approaches. Front. Cell. Infect. Microbiol. 2:138 10.3389/fcimb.2012.00138 23162800 PMC3498739

[B16] Nguyen Y. Sperandio V. (2012). Enterohemorrhagic *E. coli* (EHEC) pathogenesis. Front. Cell. Infect. Microbiol. 2:90 10.3389/fcimb.2012.00090 22919681 PMC3417627

[B17] Steyert S. R. Sahl J. W. Fraser C. M. Teel L. D. Scheutz F. Rasko D. A. (2012). Comparative genomics and stx phage characterization of LEE-negative Shiga toxin-producing *Escherichia coli*. Front. Cell. Infect. Microbiol. 2:133 10.3389/fcimb.2012.00133 23162798 PMC3491183

[B18] Tomat D. Migliore L. Aquili V. Quiberoni A. Balagué C. (2013). Phage biocontrol of enteropathogenic and shiga toxin-producing *Escherichia coli* in meat products. Front. Cell. Infect. Microbiol. 3:20 10.3389/fcimb.2013.00020 23761050 PMC3674477

